# Substance Use Prevention Programs for Indigenous Adolescents in the United States of America, Canada, Australia and New Zealand: Protocol for a Systematic Review

**DOI:** 10.2196/resprot.9012

**Published:** 2018-02-01

**Authors:** Mieke Snijder, Lexine Stapinski, Briana Lees, Nicola Newton, Katrina Champion, Catherine Chapman, James Ward, Maree Teesson

**Affiliations:** ^1^ National Drug and Alcohol Research Centre University of New South Wales Randwick Australia; ^2^ Department of Preventive Medicine Northwestern University Feinberg School of Medicine Chicago, IL United States; ^3^ South Australian Health and Medical Research Institute Adelaide Australia; ^4^ Flinders University Adelaide Australia

**Keywords:** prevention, Indigenous population, minority groups, Indians, North American, Alaska Natives, Aborigines, Australian, adolescent alcohol use, substance abuse, tobacco, marijuana smoking

## Abstract

**Background:**

Indigenous adolescents are at a higher risk of experiencing harms related to substance use compared with their non-Indigenous counterparts as a consequence of earlier onset and higher rates of substance use. Early onset of substance use has been identified as a risk factor for future substance use problems and other health, social, and family outcomes. Therefore, prevention of substance use among adolescents has been identified as a key area to improve health of Indigenous Peoples. Evidence exists for the effectiveness of prevention approaches for adolescents in mainstream populations and, most recently, for the use of computer- and Internet-delivered interventions to overcome barriers to implementation. However, there is currently no conclusive evidence about the effectiveness of these approaches for Indigenous adolescents.

**Objective:**

The purpose of this review is to synthesize the international evidence regarding the effectiveness of substance use prevention programs for Indigenous adolescents in the United States, Canada, Australia, and New Zealand.

**Methods:**

A total of 8 peer-reviewed databases and 20 gray literature databases will be searched, using search terms in line with the aims of this review and based on previous relevant reviews of substance use prevention. Studies will be included if they evaluate a substance use prevention program with Indigenous adolescents (aged 10 to 19 years) as the primary participant group and are published between January 1, 1990 and August 31, 2017.

**Results:**

A narrative synthesis will be provided about the effectiveness of the programs, the type of program (whether culture-based, adapted, or unadapted), delivery of the program (computer- and Internet-delivered or traditional), and the setting in which the programs are delivered (community, school, family, clinical, or a combination).

**Conclusions:**

The study will identify core elements of effective substance use prevention programs among Indigenous adolescents and appraise the methodological quality of the studies. This review will provide researchers, policy makers, and program developers with evidence about the potential use of prevention approaches for Indigenous adolescents.

## Introduction

### Substance Use Among Indigenous Adolescents

Indigenous people have the oldest continuing cultures in the world [1,2]. A common experience among Indigenous people is the lasting impact of colonization, which continues to impact the health and well-being of many Indigenous people today [3,4]. Combined with lower outcomes in many social determinants of health such as education, poorer access to health services, ongoing racism, and housing and employment opportunities, one of the most visible consequences is the increased susceptibility to substance use and related harms experienced by Indigenous adolescents aged 10 to 19 years. This is evident in the lower age of initiation and higher rates of use; For instance, in Australia, substance use initiation among Aboriginal and Torres Strait Islander adolescents is reported to be 2 to 6 years earlier than the national average [5], with some adolescents trying tobacco and petrol sniffing as young as 8 to 10 years old [6,7]. Additionally, Aboriginal and Torres Strait Islander adolescents are 3 times more likely to report injecting drugs [8]. American Indian and Alaskan Native adolescents, aged 14 to 15 years, are 5 times more likely to report cannabis use and more than twice as likely to report excess alcohol use, compared with their non-Indigenous counterparts [9]. A total 21% of American Indian adolescents living on reserves have tried cannabis in their lifetime, compared with 5% of non-Indigenous adolescents [10]. In Canada, Indigenous adolescents aged 12 to 18 years have been estimated to be twice as likely to report being a current smoker, compared with the non-Indigenous population [11]. Early onset of substance use among Indigenous adolescents has been identified as a risk factor for problematic substance use later in life, as well as other adverse health, social, and family outcomes [12-18]. Prevention of adolescent substance use has therefore been identified as a key strategy to improve Indigenous health [3,19].

### Potential of Web-Based Substance Use Prevention Programs

A number of prevention strategies have been developed and evaluated with the aim of delaying and reducing adolescent substance use and preventing associated harms. For mainstream populations, school-based prevention programs have been found to be highly effective in reducing the onset and escalation of substance use [20-22]. Community-based and family-based approaches show considerable promise of effectiveness [23,24], whereas mass media campaigns are deemed not effective in improving drug-related knowledge or reducing substance use [24]. Despite the availability of effective prevention strategies, these programs are not widely implemented, with time and lack of resources commonly cited as barriers to implementation [24,25]. To address these barriers, a number of programs facilitated by computers (including other electronic devices such as tablets or mobile phones) or the Internet have been developed, with promising results in mainstream populations [26-30]. Champion et al [31] systematically reviewed 9 randomized controlled trials (RCTs) of computer- and Internet-delivered prevention programs, of which 6 achieved significant benefits for drug and alcohol outcomes. Advantages of computer- and Internet-delivered prevention programs include reduced implementation costs, higher degrees of implementation fidelity, and less need for personnel to deliver the program [30,31]. Computer- and Internet-delivered prevention programs may be particularly beneficial for disenfranchised populations, such as Indigenous adolescents, because these programs can overcome issues with access, provide engagement opportunities, and have been found to be culturally compatible for Indigenous adolescents [32-35]. Moreover, recent research has shown that Internet and technology use is higher or just as high among Indigenous people, compared with non-Indigenous people, and that Indigenous adolescents feel comfortable using technology and expressing themselves on the Internet [35,36].

### Need for Evidence-Based Prevention for Indigenous Adolescents

Although there is evidence to support computer- and Internet-delivered substance use prevention approaches in mainstream populations, the effectiveness of these programs cannot be assumed for Indigenous populations. Indigenous populations may require a cross-cultural translation of these approaches, mapped against situational contexts including different communication styles, languages, and different understandings of health and identity [37-39]. This may involve adaptation of an existing mainstream program to align with cultural identity and practices (culturally adapted programs), or development of programs specifically for the local Indigenous cultural context (culture-based programs) [40]. Although it is generally accepted that prevention programs should have a good cultural fit with the local cultural context, no studies have systematically assessed whether culture-based, culturally adapted, or culturally unadapted programs are most effective. Furthermore, no conclusive evidence currently exists for the effectiveness of substance use prevention approaches for Indigenous adolescents, including evidence about the most effective type, setting, or delivery method. A recent Australian systematic review of substance use prevention for Aboriginal and Torres Strait Islander youth found limited evidence for the effectiveness of the 8 reviewed programs, primarily due to poor evaluation designs [41]. Other previous reviews have not been able to provide a comprehensive synthesis of international evidence regarding effective prevention approaches for Indigenous populations, because they have focused on one substance [42,43], one program setting [44,45], or were primarily focused on one country [43-46]. This systematic review will address this gap by reviewing the evidence regarding the effectiveness of prevention programs in reducing substance use and related outcomes for Indigenous adolescents in the United States, Canada, Australia, and New Zealand. These four countries were chosen because Indigenous people have a comparable history of colonization and dispossession by English settlers, resulting in predominantly English-speaking culture in which Indigenous people are a minority. In all the four countries, there is an unequal distribution between Indigenous and non-Indigenous people in terms of the distribution of economic, social, and health care resources. Indigenous people are more likely to live under the poverty line and are over-represented in measures of low socioeconomic position [3]. Consequently, Indigenous people in these four countries experience poorer health and social outcomes compared with their non-Indigenous counterparts [3,4,47]. Although there are differences between Indigenous Peoples’ culture between these countries and within these countries, similarities exist including an ongoing occupation of the ancestral lands; common ancestry of the occupied land; and cultural norms and values such as ancestors, connection to ancestors, country, family and community, the concept of health as being holistic, and spirituality [1].

### Aims of Literature Review

For Indigenous adolescents, this review will investigate the following: (1) the effectiveness of culturally adapted substance use prevention programs compared with culture-based or culturally unadapted programs in reducing substance use and related outcomes; (2) the effectiveness of prevention programs delivered in a school setting, compared with community, family, clinical, or multisetting (ie, school, community and/or family) in reducing substance use and related outcomes; (3) the effectiveness of computer- and Internet-delivered programs, compared with traditional delivery; (4) the elements of effective substance use prevention programs; and (5) the methodological quality of evaluations of substance use prevention programs.

## Methods

### Protocol Registration

The protocol for this systematic review has been registered in the PROSPERO registry of the University of York (registration number: CRD42017081885) and has followed the Preferred Reporting Items for Systematic Reviews and Meta-Analyses Protocol (PRISMA-P) guidelines; see Multimedia Appendix 1 [48].

### Search Strategy

Peer-reviewed and gray literature databases will be searched. Searches in the following 8 electronic databases of peer-reviewed journals will be conducted: DRUG, Cochrane, Embase, PsycINFO, Medline, ProQuest, Informit, and CINAHL. Searches of the gray literature will be conducted in the 20 databases listed in Textbox 1. These databases were based on searches conducted in previous literature reviews on topics related to the health of Indigenous Peoples [49,50] and recommendations from experts and University libraries in Australia and the United States. The reference lists of selected studies will be assessed for further relevant publications. The researchers will also solicit publications from researchers in the field.

Search terms are based on previous systematic literature reviews about Indigenous substance use programs [40,46], and computer- and Internet-delivered substance use prevention [21,31]. For the peer-reviewed databases, the search strategy will consist of combinations of keywords related to the participants (“Aboriginal,” “Torres Strait Islander,” “Indigenous,” “Australia,” “New Zealand,” “Canada,” “United States of America,” “Maori,” “First Nation,” “Inuit,” “American Indians,” “Alaskan Indians” OR “Alaska Native” and “youth,” “young,” “adolescen*” OR “teen”), type of intervention (“evaluat*,” “effect*,” “efficacy,” “review,” OR “trial” and “prevention,” “intervention” “program” “educat*”), and substance-related outcomes (“substance,” “drug,” “alcohol,” “tobacco,” “petrol,” “cannabis,” “kava,” “methamphetamine,” “MDMA,” “inhalant,” “marijuana,” “amphetamine,” “psycho stimulant,” “smok*,” “illicit drug” OR “volatile drug”). Textbox 2 outlines the detailed proposed search strategy to be used in Medline. For the gray literature databases, the search strategy will consist of combinations of keywords and/or topic headings related to the participants and the substance-related outcomes.

### Eligibility Criteria

Studies will be included if they are published in English language and evaluate a substance use prevention program with Indigenous adolescents from the United States, Canada, Australia, and New Zealand as the primary participant group. Studies will be included if they are published between January 1, 1990 and August 31, 2017. This will capture studies conducted in the early days of substance prevention as well as the most recent studies. Studies will be included in the review provided the participants are Indigenous adolescents, or a mixed sample of adolescents and adults, but with adolescents as the primary target group of the program. The World Health Organization defines adolescents as people aged 10 to 19 years [12,51]. To be eligible, at least 50% of the sample must identify as Indigenous or the study must report a separate analysis for Indigenous participants.

Studies will be included if they evaluated a prevention program. The search will not be limited to randomized controlled trials (RCTs), as previous reviews in Indigenous substance use evaluations have recorded a lack of RCTs being conducted within this population [52]. Evaluation is therefore defined as either comparing an experimental group with a control group (eg, no intervention, education as usual, or an alternate intervention) and/or comparing change in outcomes across two or more time points. Following a previous review in substance use prevention among Indigenous adolescents [46], studies will be included if the evaluated prevention program has one or more of the following aims: (1) reduce substance use; (2) increase knowledge of substances and their effects; (3) change attitudes toward substances; (4) increase substance use resistance skills; (5) delay substance use initiation; and/or (6) reduce intention to use substances. This review will include both computer- and Internet-delivered and traditional (face-to-face) prevention programs. It will include universal (everyone in the population), selective (members who are at risk of alcohol and other drug use), and indicated (individuals experiencing early signs of alcohol and other drug use) prevention programs.

### Study Record Management

All publications identified in the search of peer-reviewed databases and relevant publications from the gray literature will be exported into a bibliographic software Endnote (Clarivate Analytics, Philadelphia, PA, USA), including the citation and abstract. Duplicate publications will be removed.

**Gray literature databases included in search strategy (n=20).**

United States

- American Indian Health
- Arctic Health
- One Sky Center
- Turtle Island Native Network
- SAMHSA’s National Registry of Evidence-based Programs and Practices

Canada

- National Collaborating Centre for Aboriginal Health
- National Aboriginal Health Organization
- Indigenous Studies Portal

Australia

- The Australian Indigenous Health InfoNet
- Closing the Gap Clearinghouse
- Analysis and Policy Observatory

New Zealand

- Maori Health

International

- Global Health
- Prevention Information & Evidence
- Gray Literature Report
- PAIS Index
- Campbell Library
- Minority Health and Health Equity Archive
- CrimeSolutions
- Native Health Databases

**Search strategy for systematic review of substance use prevention programs for Indigenous adolescents (example: Medline search).**

1. ((substance OR drug OR alcohol OR tobacco OR petrol OR cannabis OR kava OR methamphetamine OR MDMA OR inhalant OR marijuana OR amphetamine OR “psycho stimulant” OR smok* OR “illicit drug” OR “volatile drug”) AND (evaluat* OR effect* OR efficacy OR review OR trial) AND ((Indigenous OR Aborigin* OR “Torres Strait*” OR Maor* OR “First Nation” OR Inuit OR “American Indian*” OR “Alaskan Indian*”) AND (Austral* OR “New Zealand*” OR Canad* OR Americ*)) AND (youth OR young OR adolescen* OR teen*)).mp. AND (educat* OR prevent* OR interven* OR program).m_titl.
2. limit 1 to yr=“1990 - 2017”

[mp=title, abstract, original title, name of substance word, subject heading word, keyword heading word, protocol supplementary concept word, rare disease supplementary concept word, unique identifier]

**Figure 1:**
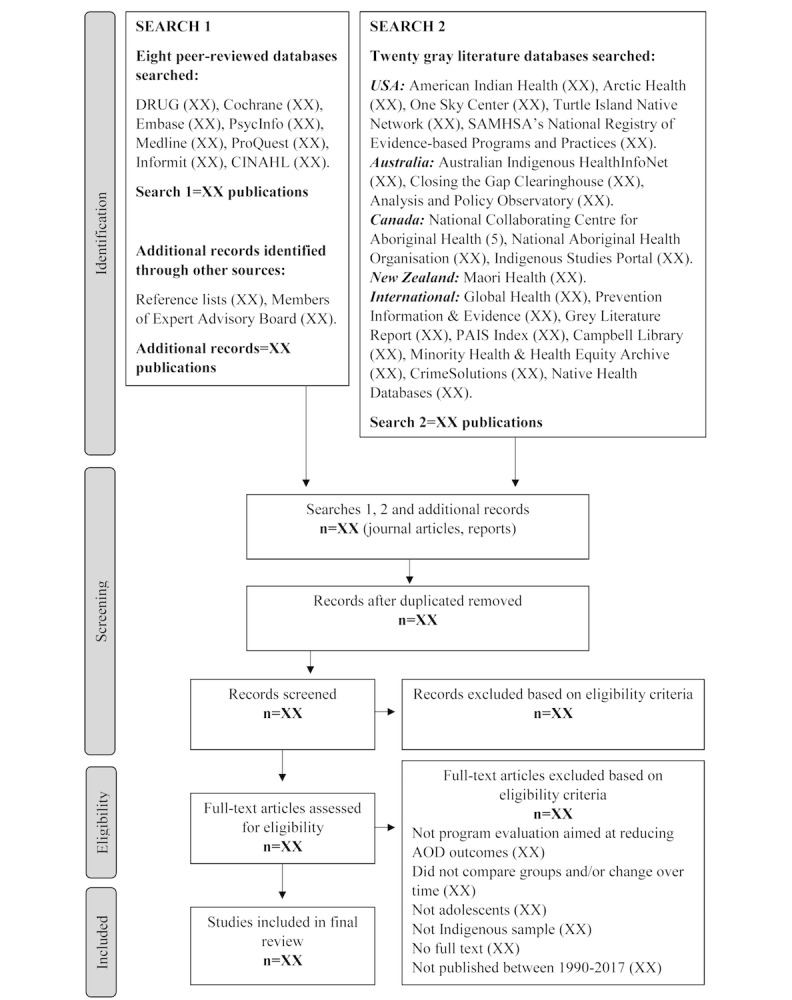
Preferred Reporting Items for Systematic Reviews and Meta-Analyses Protocol (PRISMA) flow diagram of search and selection strategy.

### Selection Processes

Figure 1 illustrates the steps to be taken in the study selection process. One reviewer (BL) will screen all titles and abstracts of papers identified in the searches and assess their eligibility against the inclusion criteria. A second reviewer (MS) will screen a random selection of 25% of the publications to ensure accuracy in the study selection. Agreement between the two reviewers will be assessed, and where there is disagreement, this will be reconciled in consultation between the two authors.

After initial screening, full text copies of the publications identified as potentially relevant will be downloaded and further assessed for their eligibility by the two reviewers (BL and MS). Cohen kappa will be calculated to evaluate the inter-rater agreement between the two reviewers at the full text screening stage. Where there is disagreement, this will be reconciled in discussion between the two authors. If there is no consensus, expert consultation will be sought from the other, more senior authors. The first reviewer (BL) will screen the reference lists of the eligible studies for further publications to be added into the systematic review.

### Data Extraction

One reviewer (BL) will extract the following data from the publications into Microsoft Excel: authors, year of publication, country, name of the evaluated program, study design, sample size, targeted age, Indigenous status of participants, target substance, geographical area, setting, type of program, intervention strategy, duration and frequency of program, whether booster sessions were provided, follow-up time points, mode of delivery, community and stakeholder involvement in development, program facilitation, language consideration, control group, outcome measures used, overall quantitative and overall qualitative outcomes related to substance use, and harms. Detailed qualitative and quantitative outcomes (substance-use related or other outcomes measured) for each study will be extracted.

Setting of the programs will be classified into school-based, community-based, family-based, clinical, or multi-setting. School-based programs are defined as those implemented either in a classroom setting during school hours, or as an out-of-school activity delivered by the school. Community-based programs are those implemented with groups within the community (ie, adolescents, parents, or whole community). Family-based are the programs targeting the family unit. Clinical settings are based within a health service, such as a community health service or a general practice. Multi-setting programs have a combination of any of the aforementioned settings.

Type of programs will be classified following recommendations by Leske et al [40] into culture-based, culturally adapted, or culturally unadapted programs. Culture-based programs are developed to reflect and incorporate the unique cultural values and beliefs of the Indigenous participants. Culturally adapted programs are modified from existing non-Indigenous programs to be more culturally appropriate to the Indigenous participants. Culturally unadapted programs are developed for other cultural groups (eg, European, African American, or Mexican) and delivered to Indigenous participants without modification.

Program delivery will be identified as computer- and Internet-delivered or traditional. Computer- and Internet-delivered programs are those that are delivered completely or partially using computers or other Web-based technology. Traditional programs are those in which no part of the program is delivered using technology.

According to the most commonly measured substance-related outcomes following Lee et al [46], this study will group outcomes as follows: substance-related knowledge, substance use, and attitudes toward substance use. These 3 outcomes capture most substance-related outcomes studied in substance use prevention programs. To identify the elements of beneficial substance use prevention programs for Indigenous adolescents, two reviewers (BL and MS) will extract the program elements from all studies to identify the key elements of prevention programs that lead to beneficial substance-related outcomes for Indigenous adolescents.

### Data Synthesis

A narrative summary will be provided of the outcomes of the included studies. On the basis of previous reviews of evaluation studies in Indigenous populations, we expect the number of studies to be too low and the quality of studies too varied to warrant a quantitative synthesis of the data [52-54]. The narrative summary will include a discussion of whether programs are beneficial to prevention of substances among Indigenous adolescents. Programs will be identified as beneficial if there are beneficial effects on more than 50% of substance-related outcomes, studies reporting positive findings on 50% or less of the evaluated outcomes will be classified as mixed, studies reporting negative outcomes will be classified as iatrogenic, and studies without significant outcomes will be classified as null. The narrative summary will discuss the number of beneficial programs for each program type (culture-based, culturally adapted, and culturally unadapted), type of delivery (computer- and Internet-delivered and traditional), and program setting (school, community, family, clinical, and multi-setting). It will also detail the elements used in the beneficial programs and summarize the most commonly implemented elements.

### Critical Appraisal of Risk of Bias in Individual Studies

The methodological quality of both quantitative and qualitative elements of the studies will be assessed. The quality assessment will be conducted by one reviewer (BL), with a second reviewer (MS) appraising a random selection of 25% of the publications to ensure reliable coding. Following previous systematic reviews of prevention programs for Indigenous people [49,55,56], this review will assess the quality of quantitative studies using the Dictionary for Effective Public Health Practice Project Quality Assessment Tool for Quantitative Studies [57]. Sections A (selection bias), B (study design), C (confounders), E (data collection), and F (withdrawals and dropouts) of this tool are rated as strong, moderate, or weak to assess possible bias. Section D (blinding) will be excluded from this study because double-blinding is not feasible in school-based or community-based studies [23]. As prescribed, sections G (intervention integrity) and H (analysis appropriateness) will receive a narrative description rather than categorical ratings. Following standard procedures of this tool, each study will receive a summary rating defined as weak (two or more weak scores), moderate (one weak score is given), or strong (no weak scores are given).

The methodological quality of qualitative study components will be assessed using a modified version of the qualitative tool by Long and Godfrey [58], which has also previously been used in a systematic review of programs for Aboriginal and Torres Strait Islander people in Australia [56]. The adapted version assesses quality in 3 domains related to evaluation: (1) data collection, the need for clear descriptions of the data collection process; (2) analysis and potential research bias, the transparency of the description of data analyses processes, description of researchers’ positioning in the study and the interpretation of findings in line with the literature ; and (3) policy and practical implications, assessment of the populations to which the findings are generalizable and implications for policy and practice.

## Results

Data analysis is underway and the results of this systematic review are expected to be submitted for publication in 2018.

## Discussion

This paper summarizes the protocol for a systematic review of substance use prevention programs for Indigenous adolescents in the United States, Canada, Australia, and New Zealand. The purpose of this review is to synthesize international evidence regarding the effectiveness of substance use prevention programs for Indigenous populations. It will identify the setting in which prevention programs are most effective, the most beneficial delivery and types of programs, and the elements of effective substance use prevention for Indigenous adolescents.

This review will provide researchers, policy makers, and program developers with up-to-date information about the strength of the international evidence to support the use of substance use prevention approaches among Indigenous adolescents. It will evaluate whether mainstream programs are effective when implemented in culturally unadapted form among Indigenous adolescents, and will assess evidence supporting the effectiveness of culturally adapted mainstream programs and specific culture-based programs. Finally, this review will provide evidence about the potential to use computer- and Internet-delivered prevention approaches among Indigenous populations.

Given the high rates of technology and Internet use amongst Indigenous adolescents [34,36] and effectiveness of computer- and Internet-delivered substance use prevention in mainstream populations [30], there is considerable potential for the use of computers and Web-based technology in the delivery of substance use prevention with Indigenous adolescents [32]. This review will inform the development of future computer- and Internet-delivered prevention programs for Indigenous adolescents, which have the potential to be highly advantageous for Indigenous adolescents, due to the sustainable, low cost, and engaging format that is well-aligned to the preferences of adolescents [34,59].

## Appendix

Multimedia Appendix 1 Preferred Reporting Items for Systematic Reviews and Meta-Analyses Protocol (PRISMA-P) checklist.

## Abbreviations

PRISMA-P: Preferred Reporting Items for Systematic Reviews and Meta-Analyses Protocol
